# Dibromido(1,10-phenanthroline-κ^2^ *N*,*N*′)palladium(II)

**DOI:** 10.1107/S160053680905168X

**Published:** 2009-12-04

**Authors:** Kwang Ha

**Affiliations:** a School of Applied Chemical Engineering, The Research Institute of Catalysis, Chonnam National University, Gwangju 500-757, Republic of Korea

## Abstract

In the title complex, [PdBr_2_(C_12_H_8_N_2_)], the Pd^II^ ion is four-coordinated in a slightly distorted square-planar environment by two N atoms of the chelating 1,10-phenanthroline ligand and two bromide ions. The complex displays numerous inter­molecular π–π inter­actions between adjacent six-membered rings, the shortest centroid–centroid distance being 3.680 (4) Å. The nearly planar [maximum deviation 0.143 (2) Å] mol­ecules stack in columns parallel to (101) with a Pd⋯Pd distance of 4.8466 (9) Å.

## Related literature

For the syntheses of [Pd*X*_2_(phen)] complexes (phen = 1,10-phenanthroline; *X* = Cl, Br, I or SCN), see: Cheng *et al.* (1977 ▶). For the crystal structure of yellow [PtCl_2_(phen)] which is isotypic to the title complex, see: Grzesiak & Matzger (2007 ▶). For the crystal structures of related Pd-bipy complexes, [Pd*X*_2_(bipy)] (bipy = 2,2′-bipyridine; *X* = Cl, Br or I), see: Maekawa *et al.* (1991 ▶); Smeets *et al.* (1997 ▶); Ha (2009 ▶).
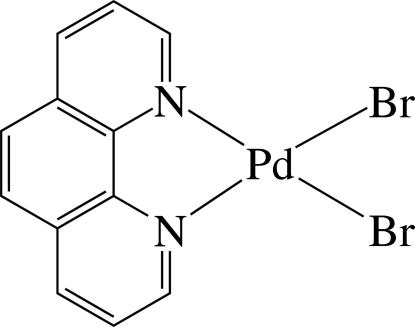


## Experimental

### 

#### Crystal data

[PdBr_2_(C_12_H_8_N_2_)]
*M* *_r_* = 446.42
Monoclinic, 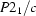

*a* = 9.9099 (6) Å
*b* = 17.4897 (10) Å
*c* = 7.2598 (4) Å
β = 109.106 (1)°
*V* = 1188.96 (12) Å^3^
*Z* = 4
Mo *K*α radiation
μ = 8.26 mm^−1^
*T* = 200 K
0.22 × 0.06 × 0.04 mm

#### Data collection

Bruker SMART 1000 CCD diffractometer
Absorption correction: multi-scan (*SADABS*; Bruker, 2000 ▶) *T*_min_ = 0.420, *T*_max_ = 0.719
8695 measured reflections
2933 independent reflections
1729 reflections with *I* > 2σ(*I*)
*R*_int_ = 0.082

#### Refinement

*R*[*F* ^2^ > 2σ(*F* ^2^)] = 0.045
*wR*(*F* ^2^) = 0.091
*S* = 1.00
2933 reflections
154 parameters
H-atom parameters constrained
Δρ_max_ = 1.37 e Å^−3^
Δρ_min_ = −1.54 e Å^−3^

### 

Data collection: *SMART* (Bruker, 2000 ▶); cell refinement: *SAINT* (Bruker, 2000 ▶); data reduction: *SAINT*; program(s) used to solve structure: *SHELXS97* (Sheldrick, 2008 ▶); program(s) used to refine structure: *SHELXL97* (Sheldrick, 2008 ▶); molecular graphics: *ORTEP-3* (Farrugia, 1997 ▶) and *PLATON* (Spek, 2009 ▶); software used to prepare material for publication: *SHELXL97*.

## Supplementary Material

Crystal structure: contains datablocks global, I. DOI: 10.1107/S160053680905168X/xu2703sup1.cif

Structure factors: contains datablocks I. DOI: 10.1107/S160053680905168X/xu2703Isup2.hkl

Additional supplementary materials:  crystallographic information; 3D view; checkCIF report

## Figures and Tables

**Table 1:** Selected bond lengths (Å)

Pd1—N1	2.059 (6)
Pd1—N2	2.048 (6)
Pd1—Br1	2.4095 (9)
Pd1—Br2	2.4016 (10)

**Fig. 1.**
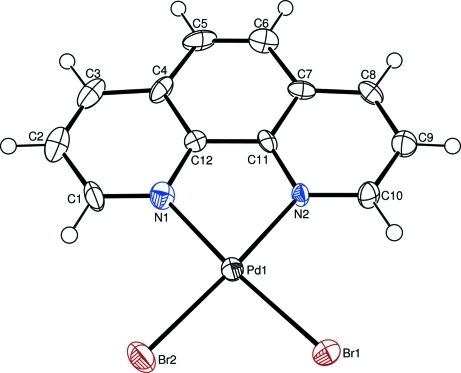
The structure of the title complex, with displacement ellipsoids drawn at the 50% probability level for non-H atoms.

**Fig. 2.**
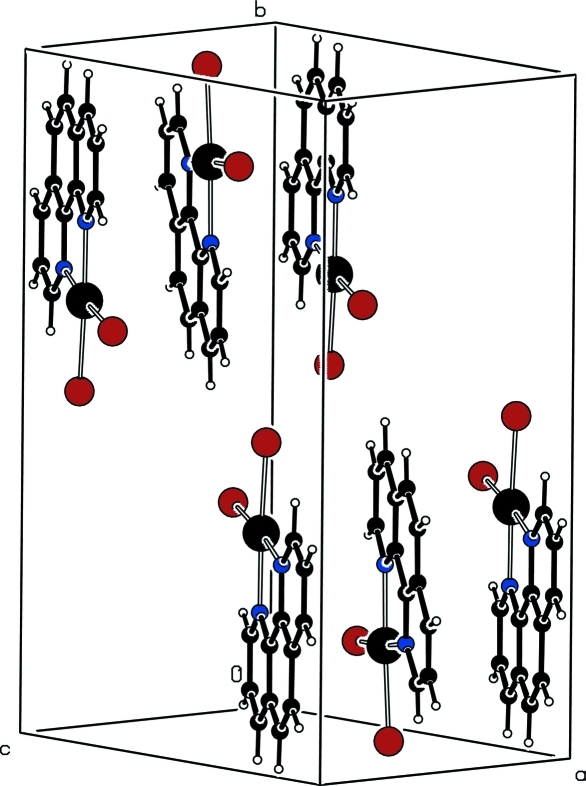
Crystal packing of the title complex.

## supplementary crystallographic information

### Comment

The title complex, [PdBr_2_(phen)] (where phen is 1,10-phenanthroline, C_12_H_8_N_2_), is isomorphous with the yellow form of [PtCl_2_(phen)], whereas the orange form of [PtCl_2_(phen)] crystallized in the orthorhombic space group *Pca*2_1_ (Grzesiak & Matzger, 2007).

In the title complex, the Pd^2+^ ion is four-coordinated in a slightly distorted square-planar environment by two N atoms of the chelating 1,10-phenanthroline ligand and two bromide ions (Fig. 1). The main contribution to the distortion is the tight N1—Pd1—N2 chelate angle [81.5 (2)°], which results in non-linear *trans* arrangement [<N1—Pd1—Br1 = 175.29 (19)° and <N2—Pd1—Br2 = 175.30 (15)°]. The Pd1—N and Pd1—Br bond lengths are almost equal, respectively [Pd1—N: 2.059 (6) and 2.048 (6) Å; Pd1—Br 2.4095 (9) and 2.4016 (10) Å]. The complex displays numerous intermolecular π-π interactions between adjacent six-membered rings, with a shortest centroid-centroid distance of 3.680 (4) Å and the dihedral angle between the ring planes is 5.0 (4)°. The nearly planar [PdBr_2_(phen)] molecules stack columnarly parallel to the (101) plane with a Pd···Pd distance of 4.8466 (9) Å (Fig. 2).

### Experimental

To a solution of K_2_PdBr_4_ (0.2033 g, 0.403 mmol) in H_2_O (20 ml) was added 1,10-phenanthroline (0.0727 g, 0.403 mmol) and refluxed for 3 h. The precipitate obtained was separated by filtration, washed with water and acetone, and dried at 70 °C, to give a yellow powder (0.1420 g). Crystals suitable for X-ray analysis were obtained by slow evaporation from an ethanol solution.

### Refinement

H atoms were positioned geometrically and allowed to ride on their respective parent atoms [C—H = 0.95 Å and *U*_iso_(H) = 1.2*U*_eq_(C)].

### Crystal data

**Table:**

[PdBr_2_(C_12_H_8_N_2_)]	*F*(000) = 840
*M**_r_* = 446.42	*D*_x_ = 2.494 Mg m^−^^3^
Monoclinic, *P*2_1_/*c*	Mo *K*α radiation, λ = 0.71073 Å
Hall symbol: -P 2ybc	Cell parameters from 1837 reflections
*a* = 9.9099 (6) Å	θ = 2.3–28.2°
*b* = 17.4897 (10) Å	µ = 8.26 mm^−^^1^
*c* = 7.2598 (4) Å	*T* = 200 K
β = 109.106 (1)°	Needle, yellow
*V* = 1188.96 (12) Å^3^	0.22 × 0.06 × 0.04 mm
*Z* = 4	

### Data collection

**Table:**

Bruker SMART 1000 CCD diffractometer	2933 independent reflections
Radiation source: fine-focus sealed tube	1729 reflections with *I* > 2σ(*I*)
graphite	*R*_int_ = 0.082
φ and ω scans	θ_max_ = 28.3°, θ_min_ = 2.2°
Absorption correction: multi-scan (*SADABS*; Bruker, 2000)	*h* = −13→12
*T*_min_ = 0.420, *T*_max_ = 0.719	*k* = −23→23
8695 measured reflections	*l* = −9→9

### Refinement

**Table:**

Refinement on *F*^2^	Primary atom site location: structure-invariant direct methods
Least-squares matrix: full	Secondary atom site location: difference Fourier map
*R*[*F*^2^ > 2σ(*F*^2^)] = 0.045	Hydrogen site location: inferred from neighbouring sites
*wR*(*F*^2^) = 0.091	H-atom parameters constrained
*S* = 1.00	*w* = 1/[σ^2^(*F*_o_^2^) + (0.0152*P*)^2^] where *P* = (*F*_o_^2^ + 2*F*_c_^2^)/3
2933 reflections	(Δ/σ)_max_ < 0.001
154 parameters	Δρ_max_ = 1.37 e Å^−^^3^
0 restraints	Δρ_min_ = −1.54 e Å^−^^3^

### Special details

**Table:**

Geometry. All e.s.d.'s (except the e.s.d. in the dihedral angle between two l.s. planes) are estimated using the full covariance matrix. The cell e.s.d.'s are taken into account individually in the estimation of e.s.d.'s in distances, angles and torsion angles; correlations between e.s.d.'s in cell parameters are only used when they are defined by crystal symmetry. An approximate (isotropic) treatment of cell e.s.d.'s is used for estimating e.s.d.'s involving l.s. planes.
Refinement. Refinement of *F*^2^ against ALL reflections. The weighted *R*-factor *wR* and goodness of fit *S* are based on *F*^2^, conventional *R*-factors *R* are based on *F*, with *F* set to zero for negative *F*^2^. The threshold expression of *F*^2^ > σ(*F*^2^) is used only for calculating *R*-factors(gt) *etc*. and is not relevant to the choice of reflections for refinement. *R*-factors based on *F*^2^ are statistically about twice as large as those based on *F*, and *R*- factors based on ALL data will be even larger.

### Fractional atomic coordinates and isotropic or equivalent isotropic displacement parameters (Å^2^)

**Table:**

	*x*	*y*	*z*	*U*_iso_*/*U*_eq_	
Pd1	0.70926 (6)	0.34181 (3)	−0.10593 (9)	0.02059 (16)	
Br1	0.74510 (9)	0.47817 (4)	−0.08022 (13)	0.0299 (2)	
Br2	0.46176 (9)	0.36087 (5)	−0.29008 (13)	0.0352 (2)	
N1	0.6966 (7)	0.2243 (3)	−0.1180 (9)	0.0251 (15)	
N2	0.9155 (6)	0.3173 (3)	0.0591 (8)	0.0172 (13)	
C1	0.5885 (8)	0.1797 (4)	−0.2101 (11)	0.0285 (19)	
H1	0.5012	0.2027	−0.2859	0.034*	
C2	0.5963 (10)	0.1002 (4)	−0.2012 (12)	0.036 (2)	
H2	0.5169	0.0698	−0.2723	0.043*	
C3	0.7203 (9)	0.0668 (4)	−0.0885 (12)	0.033 (2)	
H3	0.7257	0.0127	−0.0787	0.040*	
C4	0.8411 (9)	0.1110 (4)	0.0142 (12)	0.0251 (19)	
C5	0.9779 (9)	0.0822 (4)	0.1358 (12)	0.032 (2)	
H5	0.9912	0.0286	0.1554	0.039*	
C6	1.0848 (9)	0.1291 (4)	0.2199 (11)	0.0277 (19)	
H6	1.1740	0.1079	0.2954	0.033*	
C7	1.0718 (8)	0.2103 (4)	0.2022 (11)	0.0217 (17)	
C8	1.1806 (8)	0.2624 (4)	0.2872 (11)	0.0249 (18)	
H8	1.2718	0.2449	0.3664	0.030*	
C9	1.1558 (8)	0.3383 (4)	0.2568 (12)	0.0297 (19)	
H9	1.2298	0.3739	0.3152	0.036*	
C10	1.0228 (8)	0.3643 (4)	0.1406 (11)	0.0226 (18)	
H10	1.0085	0.4177	0.1190	0.027*	
C11	0.9395 (7)	0.2402 (4)	0.0878 (10)	0.0167 (16)	
C12	0.8238 (8)	0.1901 (4)	−0.0051 (10)	0.0188 (17)	

### Atomic displacement parameters (Å^2^)

**Table:**

	*U*^11^	*U*^22^	*U*^33^	*U*^12^	*U*^13^	*U*^23^
Pd1	0.0183 (3)	0.0196 (3)	0.0226 (3)	−0.0008 (3)	0.0051 (2)	0.0000 (3)
Br1	0.0289 (5)	0.0197 (4)	0.0388 (5)	0.0016 (4)	0.0078 (4)	0.0009 (4)
Br2	0.0193 (4)	0.0391 (5)	0.0408 (6)	0.0006 (4)	0.0013 (4)	0.0039 (4)
N1	0.026 (4)	0.026 (3)	0.026 (4)	−0.005 (3)	0.012 (3)	0.001 (3)
N2	0.014 (3)	0.016 (3)	0.018 (3)	−0.003 (3)	−0.001 (3)	0.001 (3)
C1	0.015 (4)	0.039 (5)	0.027 (5)	−0.007 (4)	0.001 (3)	−0.005 (4)
C2	0.046 (6)	0.032 (5)	0.029 (5)	−0.014 (5)	0.011 (4)	−0.008 (4)
C3	0.053 (6)	0.021 (4)	0.034 (5)	−0.010 (4)	0.024 (5)	−0.008 (4)
C4	0.038 (5)	0.014 (4)	0.030 (5)	−0.009 (4)	0.020 (4)	−0.003 (3)
C5	0.047 (6)	0.015 (4)	0.042 (6)	0.013 (4)	0.025 (5)	0.011 (4)
C6	0.032 (5)	0.028 (4)	0.026 (5)	0.007 (4)	0.012 (4)	0.011 (4)
C7	0.023 (4)	0.025 (4)	0.022 (4)	0.008 (4)	0.014 (4)	0.004 (4)
C8	0.014 (4)	0.033 (5)	0.024 (5)	0.005 (4)	0.001 (3)	0.004 (4)
C9	0.025 (5)	0.025 (4)	0.036 (5)	0.000 (4)	0.006 (4)	0.002 (4)
C10	0.024 (5)	0.019 (4)	0.026 (5)	−0.005 (3)	0.009 (4)	0.004 (3)
C11	0.012 (4)	0.025 (4)	0.016 (4)	−0.004 (3)	0.009 (3)	0.000 (3)
C12	0.020 (4)	0.018 (4)	0.018 (4)	0.000 (3)	0.007 (3)	0.001 (3)

### Geometric parameters (Å, °)

**Table:**

Pd1—N1	2.059 (6)	C4—C12	1.395 (9)
Pd1—N2	2.048 (6)	C4—C5	1.445 (11)
Pd1—Br1	2.4095 (9)	C5—C6	1.321 (10)
Pd1—Br2	2.4016 (10)	C5—H5	0.9500
N1—C1	1.317 (9)	C6—C7	1.428 (9)
N1—C12	1.394 (9)	C6—H6	0.9500
N2—C10	1.321 (8)	C7—C8	1.392 (10)
N2—C11	1.373 (8)	C7—C11	1.404 (10)
C1—C2	1.392 (10)	C8—C9	1.355 (9)
C1—H1	0.9500	C8—H8	0.9500
C2—C3	1.365 (11)	C9—C10	1.388 (10)
C2—H2	0.9500	C9—H9	0.9500
C3—C4	1.414 (11)	C10—H10	0.9500
C3—H3	0.9500	C11—C12	1.426 (9)
			
N2—Pd1—N1	81.5 (2)	C6—C5—C4	121.0 (7)
N2—Pd1—Br2	175.30 (15)	C6—C5—H5	119.5
N1—Pd1—Br2	94.47 (19)	C4—C5—H5	119.5
N2—Pd1—Br1	93.91 (15)	C5—C6—C7	122.8 (8)
N1—Pd1—Br1	175.29 (19)	C5—C6—H6	118.6
Br2—Pd1—Br1	90.20 (3)	C7—C6—H6	118.6
C1—N1—C12	118.2 (6)	C8—C7—C11	117.1 (6)
C1—N1—Pd1	129.9 (6)	C8—C7—C6	125.3 (7)
C12—N1—Pd1	111.9 (5)	C11—C7—C6	117.6 (8)
C10—N2—C11	118.0 (6)	C9—C8—C7	119.8 (7)
C10—N2—Pd1	129.4 (5)	C9—C8—H8	120.1
C11—N2—Pd1	112.6 (4)	C7—C8—H8	120.1
N1—C1—C2	123.0 (8)	C8—C9—C10	120.4 (8)
N1—C1—H1	118.5	C8—C9—H9	119.8
C2—C1—H1	118.5	C10—C9—H9	119.8
C3—C2—C1	118.7 (8)	N2—C10—C9	122.2 (7)
C3—C2—H2	120.6	N2—C10—H10	118.9
C1—C2—H2	120.6	C9—C10—H10	118.9
C2—C3—C4	121.5 (7)	N2—C11—C7	122.5 (7)
C2—C3—H3	119.3	N2—C11—C12	117.3 (6)
C4—C3—H3	119.3	C7—C11—C12	120.1 (7)
C12—C4—C3	115.7 (8)	N1—C12—C4	122.8 (7)
C12—C4—C5	117.9 (7)	N1—C12—C11	116.6 (6)
C3—C4—C5	126.4 (7)	C4—C12—C11	120.6 (7)
			
N2—Pd1—N1—C1	−178.2 (7)	Pd1—N2—C10—C9	176.9 (5)
Br2—Pd1—N1—C1	4.2 (6)	C8—C9—C10—N2	1.3 (11)
N2—Pd1—N1—C12	3.5 (5)	C10—N2—C11—C7	1.2 (9)
Br2—Pd1—N1—C12	−174.1 (4)	Pd1—N2—C11—C7	−177.7 (5)
N1—Pd1—N2—C10	177.9 (6)	C10—N2—C11—C12	−178.5 (6)
Br1—Pd1—N2—C10	−1.2 (6)	Pd1—N2—C11—C12	2.6 (7)
N1—Pd1—N2—C11	−3.4 (5)	C8—C7—C11—N2	−0.2 (10)
Br1—Pd1—N2—C11	177.5 (4)	C6—C7—C11—N2	−179.3 (6)
C12—N1—C1—C2	−0.7 (11)	C8—C7—C11—C12	179.5 (6)
Pd1—N1—C1—C2	−178.9 (5)	C6—C7—C11—C12	0.3 (10)
N1—C1—C2—C3	1.6 (12)	C1—N1—C12—C4	0.0 (10)
C1—C2—C3—C4	−1.7 (12)	Pd1—N1—C12—C4	178.5 (5)
C2—C3—C4—C12	1.0 (11)	C1—N1—C12—C11	178.3 (6)
C2—C3—C4—C5	−179.3 (7)	Pd1—N1—C12—C11	−3.2 (7)
C12—C4—C5—C6	−2.5 (11)	C3—C4—C12—N1	−0.1 (10)
C3—C4—C5—C6	177.8 (7)	C5—C4—C12—N1	−179.8 (6)
C4—C5—C6—C7	2.0 (11)	C3—C4—C12—C11	−178.3 (6)
C5—C6—C7—C8	−179.9 (7)	C5—C4—C12—C11	2.0 (10)
C5—C6—C7—C11	−0.9 (11)	N2—C11—C12—N1	0.4 (9)
C11—C7—C8—C9	−0.3 (10)	C7—C11—C12—N1	−179.2 (6)
C6—C7—C8—C9	178.8 (7)	N2—C11—C12—C4	178.7 (6)
C7—C8—C9—C10	−0.3 (11)	C7—C11—C12—C4	−0.9 (10)
C11—N2—C10—C9	−1.8 (10)		

## References

[bb1] Bruker (2000). *SADABS*, *SMART* and *SAINT* Bruker AXS Inc., Madison, Wisconsin, USA.

[bb2] Cheng, C. P., Plankey, B., Rund, J. V. & Brown, T. L. (1977). *J. Am. Chem. Soc.* **99**, 8413–8417.

[bb3] Farrugia, L. J. (1997). *J. Appl. Cryst.* **30**, 565.

[bb4] Grzesiak, A. L. & Matzger, A. J. (2007). *Inorg. Chem.* **46**, 453–457. 10.1021/ic061323k 17279824

[bb5] Ha, K. (2009). *Acta Cryst.* E**65**, m1588. 10.1107/S1600536809047771 PMC2971767 21578618

[bb6] Maekawa, M., Munakata, M., Kitagawa, S. & Nakamura, M. (1991). *Anal. Sci.* **7**, 521–522.

[bb7] Sheldrick, G. M. (2008). *Acta Cryst.* A**64**, 112–122. 10.1107/S0108767307043930 18156677

[bb8] Smeets, W. J. J., Spek, A. L., Hoare, J. L., Canty, A. J., Hovestad, N. & van Koten, G. (1997). *Acta Cryst.* C**53**, 1045–1047.

[bb9] Spek, A. L. (2009). *Acta Cryst.* D**65**, 148–155. 10.1107/S090744490804362X PMC2631630 19171970

